# Non-Invasive On–Off Fluorescent Biosensor for Endothelial Cell Detection

**DOI:** 10.3390/bios14100489

**Published:** 2024-10-09

**Authors:** Qingyun Jiang, Shuai Shao, Na Li, Zhengyao Zhang, Bo Liu

**Affiliations:** 1 Cancer Hospital of Dalian University of Technology, Shenyang 110042, China; jiangqingyun@mail.dlut.edu.cn (Q.J.);; 2 Faculty of Medicine, Liaoning Key Lab of Integrated Circuit and Biomedical Electronic System, Dalian University of Technology, Dalian 116024, China; 3 School of Chemical Engineering, Ocean and Life Sciences, Dalian University of Technology, Panjin 124221, China

**Keywords:** endothelial cells (ECs), genetically encoded fluorescent protein-based biosensors (FPBs), non-invasive, phage display, affinity peptide

## Abstract

For rapid and convenient detection of living endothelial cells (ECs) specifically without immunostaining, we developed a biosensor based on turn-on fluorescent protein, named LV-EcpG. It includes a high-affinity peptide E12P obtained through phage display technology for specifically recognizing ECs and a turn-on EGFP fused with two linker peptides. The “on-off” switching mechanism of this genetically encoded fluorescent protein-based biosensor (FPB) ensured that fluorescence signals were activated only when binding with ECs, thus enabling these FPB characters for direct, visual, and non-invasive detection of ECs. Its specificity and multicolor imaging capability established LV-EcpG as a powerful tool for live EC research, with significant potential for diagnosing and treating cardiovascular diseases and tumor angiogenesis.

## 1. Introduction

Endothelial cells (ECs), as a major component of the vascular lining, play a key role in maintaining vascular health and function [1,2]. They are not only involved in the regulation of angiogenesis and blood flow but also play important roles in inflammatory responses and vascular permeability. ECs have a wide range of applications in various fields such as regenerative medicine, tissue engineering, disease modeling, and drug screening [3,4,5]. Accurate characterization ensures that the cells used have the correct phenotype and function, thus providing reliable data in research and achieving the desired effect in therapy. The identification of ECs includes immunophenotypic analysis (surface markers, CD31, CD144, von Willebrand factor (vWF)), functional testing (angiogenic capacity), and observation of cell morphology [6]. Among them, the most prominent immunophenotypic identification is commonly based on antigen–antibody immunoreactivity (immunofluorescence and flow cytometry). As such, they suffer from cumbersome labeling processes, high costs, low sensitivity, and more importantly, lack of real-time monitoring capabilities. In contrast, fluorescent biosensors offer a fast, sensitive solution that can be monitored in real time [7,8]. The currently used small molecule cell dyes such as CM-Dil [9] and Sudan III [10] are not only cytotoxic but also do not have the ability to specifically label cells.Therefore, developing a biocompatible, specific, and efficient fluorescent biosensor to overcome these limitations is a challenge.

Genetically encoded fluorescent protein-based biosensors (FPBs) have attracted much attention due to their unique advantages of good biocompatibility and the ability to bind to specific molecules for specific detection [11,12]. Among them, turn-on FPBs without ligands cannot emit light or have weak fluorescence due to the introduction of new structures in their design (off state). On the contrary, the on state is a stage where ligand binding creates an environment conducive to chromophore luminescence and enhances fluorescence signals [13], which makes turn-on FPBs particularly suitable for applications that require quantitative detection of ligand concentration with high sensitivity [14]. Of particular importance is the non-invasive and highly biocompatible detection capability of the turn-on FPBs at the live cell level, which provides a potential solution to the aforementioned limitations.

Turn-on FPBs rely on the fusion of fluorescent proteins with specific recognition domains (calcium-binding domains [15,16], metabolite-binding domains [17]). When these recognition domains bind to ligands (target molecules), they affect the fluorescence properties of the sensor, resulting in a transition from “off” to “on” [13]. Therefore, choosing an appropriate recognition domain is crucial for constructing an efficient turn-on FPB. The application of phage display technology provides a new method for identifying and screening cell surface markers [18,19], which helps to obtain peptides with high specificity and affinity. This method is expected to solve the problem of dependence on multiple biomarkers in current cell recognition and detection and help develop efficient non-invasive biosensors for ECs.

In this study, we employ phage display technology to identify a peptide, E12P, with high specificity for ECs. The peptide is inserted near the chromophore of EGFP, producing a turn-on FPB named LV-EcpG. Upon binding to ECs, conformational changes in the structural domains of LV-EcpG increase the fluorescence intensity, thus realizing the “on”–“off” state of fluorescence. Meanwhile, in vitro assays are conducted to validate the fluorescent properties, binding affinity, and specificity of the LV-EcpG biosensor. Furthermore, we explore its application in monitoring the differentiation process of mesenchymal stem cells (MSCs) into ECs. Overall, the development and application of the LV-EcpG biosensor fully demonstrate the extensive potential of genetically encoded fluorescent protein technology in the field of cell detection.

## 2. Materials and Methods

### 2.1. Phage Display Biopanning for Affinity Peptide Selection

Affinity peptide selection through phage display biopanning involved the identification of peptides that bound to a specific target. A commercial peptide phage display library (Ph.D.™-12 Phage Display Peptide Library, NEB, Ipswich, MA, USA) was employed. Endothelial cells (ECs), MSCs, and chondrocytes from ATCC were utilized in the fifth generation. To ensure specificity, subtractive negative screening was implemented. Initially, the library ($10^{12}$ PFU) was incubated with ECs ($10^{6}$) for 1 h. Subsequently, the supernatant was used for incubation with MSCs ($10^{6}$) and chondrocytes ($10^{6}$) to exclude nonspecific interactions. The eluted phages underwent titration and purification as per the manufacturer’s protocol, completing the first round of screening. Subsequent rounds of selection were conducted until the optimal results were achieved, with experiments repeated for validation. Recovery efficiency was calculated by dividing the output phage titer by the input phage titer and expressed in phage formation units (PFU).

### 2.2. ELISA

MSCs and ECs were inoculated in 96-well plates at a concentration of $10^{4}$ cells per well. After the cells reached 80%, they were treated with 4% paraformaldehyde (Solarbio, Beijing, China) for 15 min and closed with 1h containing 2.5% (*w*/*v*) bovine serum albumin (BSA) purchased from Solarbio. Subsequently, phage ($10^{12}$ PFU) was used for 1 h. Primary antibody anti-M13 (Bioss, Beijing, China) and secondary antibody HRP-mouse (Bioss, Beijing, China) were incubated for 1 h. Then, a TEM two-component color development kit (Solarbio, Beijing, China) was used. The enzyme marker (INFINITE E PLEX, TECAN, Switzerland) was read at 450nm. Randomly picked blue spots of the phage proto library were used as control, and P/N > 2 was considered positive.

### 2.3. Plasmid Construction and Protein Expression

The DNA fragments of LV-EcpG was fused into the pRSET-B vector and synthesized by Sangon Biotech. The LV-EcpG biosensors were expressed in *E. coli* BL21 (DE3) cells and induced with 0.3mM isopropyl β-D-thiogalactopyranoside for 3–4 h at 37 °C. All sensors were purified by means of nickel beads (Solarbio, Beijing, China) according to the manufacturer’s instructions. The LV-EcpG biosensor was added to the experimental buffer (20mM Tris, pH 7.5, 0.15M NaCl, 0.005% Tween-20), proteins were stored at −20 °C, and experiments were started immediately.

### 2.4. Cell Culture and Cell Differentiation

MSCs, ECs, and chondrocytes were purchased from ATCC. MSCs from p5 were seeded at $10^{5}$ cells per well and cultured in α-MEM supplemented with 10% FBS at 37 °C in a humidified atmosphere of 95% air and 5% ${\mathrm{CO}}_{2}$. Once the cells adhered to the surface, the medium was changed to endothelial cell culture medium (EBM-2) purchased from LONZA, Basel, Switzerland, with 10 mg/L VEGF and 5 mg/L bFGF (all purchased from Novoprotein, Suzhou, China) to induce MSCs to differentiate into ECs.

### 2.5. In Vitro Characterization of LV-EcpG Biosensor

We stored the purified proteins at −20 °C until they were used. The excitation range of LV-EcpG (excitation: 495±5nm; emission: 515±5nm) was from 450–500 nm, with an emission spectrum range of 500–650 nm, and readings were taken every 5 nm. Fluorescence was measured using an INFINITE E PLEX fluorescent microplate reader manufactured by TECAN. The cells (MSCs, ECs, chondrocytes) were seeded in black clear-bottom 96-well plates (JingAn Biological, Shanghai, China). In the absence of cells, control fluorescence values for LV-EcpG biosensor were measured. After incubating with LV-EcpG biosensor for 1 h, the fluorescence intensity was read.

### 2.6. Immunofluorescence

The target cells tested underwent fixation with 4% paraformaldehyde for 10 min, followed by incubation in 2% BSA at room temperature for 1 h for sealing. Subsequently, the primary antibody incubation occurred overnight at 4 °C, succeeded by secondary antibody incubation at room temperature for 1 h. The primary antibodies utilized included LV-EcpG 50μM and anti-vWF (Abcam, Cambridge, UK, 1:1000). RITC-coupled secondary antibodies against mice and rabbits from Abclonal were employed. The fluorescence excitation waves are 488 nm and 580 nm, respectively. For nuclear staining, the specimens were treated with DAPI (Solarbio).

### 2.7. Confocal Microscopy

The fluorescence images were acquired using confocal laser scanning microscopes (Olympus, Tokyo, Japan, FV3000, and Zeiss, Oberkochen, Germany, LSM980). The fluorescence excitation waves are 488 nm. The quantification of cells was performed in fields of view (FOV) measuring 400×400μm (used for counting, quantifying the relative intensity of immunofluorescence). ImageJ 1.45 software (National Institutes of Health, Bethesda, MD, USA) was used for quantification of the indicated regions in the scanned images.

### 2.8. Flow Cytometry Analysis

The LV-EcpG biosensors were labeled ECs. After incubating for 1 h, antibody labeling was performed by incubating target cells with FITC-conjugated anti-vWF (abcam, 1:100) at 4 °C for 0.5 h. The cells were fixed with 4% paraformaldehyde for 10 min. The target cells were collected, dissociated into single-cell suspensions, and filtered through a 40 μm nylon mesh. The analysis was conducted using the Accuri C6 Plus cell sorter (BD, Franklin Lakes, NJ, USA) and FlowJo™ v10.8 Software (BD Life Sciences).

## 3. Results and Discussion

### 3.1. Design Strategies for LV-EcpG Biosensor

**Figure 1:**
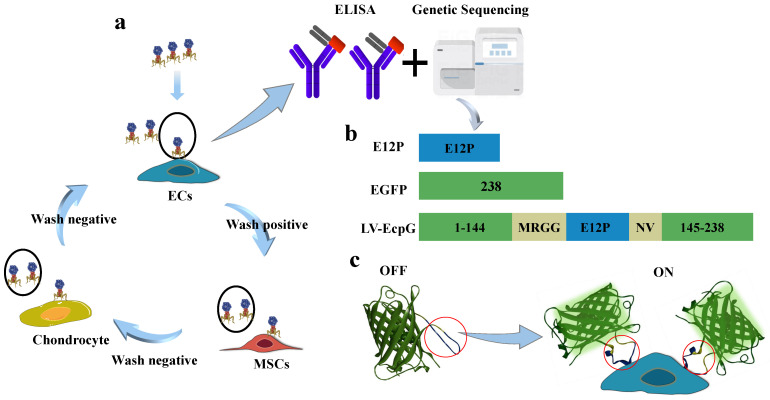
Schematic drawing of LV-EcpG biosensor. (**a**) Subtractive phage display schematic, where positive cells were identified as ECs, while MSCs and chondrocytes served as negative controls, and underwent three rounds of selection. Subsequently, the affinity peptide E12P was obtained through ELISA and gene sequencing. (**b**) Drawings for EGFP and E12P resultant LV-EcpG. (**c**) The simulation display of the LV-EcpG testing agency shows that the lock represents LV-EcpG, ECs represent the key, and ECs are displayed as the specific key for LV-EcpG to open (left). A schematic diagram of the real application of LV-EcpG, wherein the biosensor is introduced into target cells for detection. The process of fluorescence display from “off” to “on”. Conformational change domain (red circle). 3D utilizing EGFP (PDB: 6YLQ).

**Figure A1:**
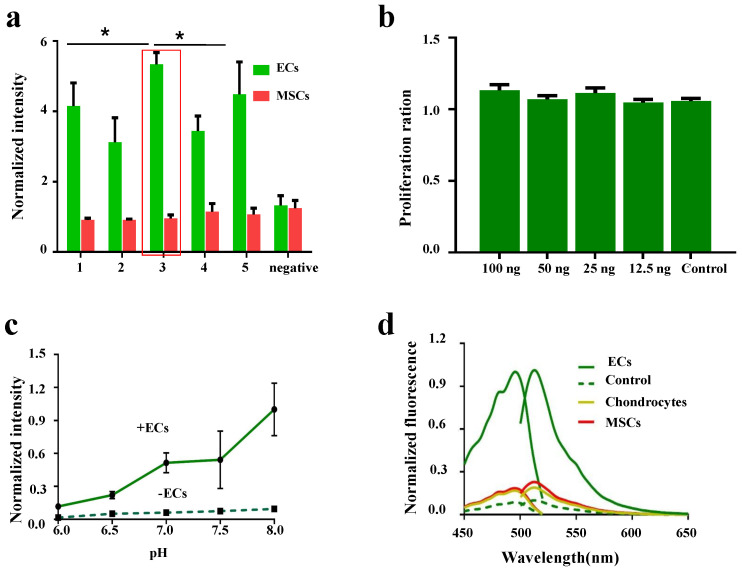
(**a**) ELISA for affinity peptides to ECs. (**b**) Effect of LV-EcpG biosensor on proliferation of ECs. (**c**) Fluorescence intensities of LV-EcpG at the indicated pH. (**d**) The excitation and emission spectra of LV-EcpG biosensor were characterized, delineating trajectories in the presence (solid lines) and absence (dashed lines) of cells. Specifically, the cells examined comprised MSCs, ECs, and chondrocytes. Each data point represents a visual field, * p<0.05, *n* = 3 technical repeats. Data are plotted as mean ± s.e.m.

Given the diverse biomarker properties expressed by ECs, establishing their specific and unique recognition molecules remains a central challenge. Developing the EC indication system based on genetic coding technology was also crucial. In this study, we employed the subtractive screening strategy of phage display technology and conducted three rounds of rigorous elution processes on purified ECs. The subsequent validation was performed by enzyme-linked immunosorbent assay (ELISA), and an affinity peptide, E12P (ANTHSNSPARGL), was successfully screened and identified that was highly specific for ECs (Figure 1a) (Figure A1a).

Based on the previous research in the field of activated genetically encoded turn-on FPBs [20,21], we engineered an innovative fluorescent biosensor through the strategic incorporation of the high-affinity peptide E12P into a variant of green fluorescent protein (GFP), followed by its fusion with EGFP. This method generated a biosensor protein, which we named LV-EcpG (Figure 1b), and successfully expressed it. This strategy aimed to visualize the energy changes caused by the binding of E12P to targeted ECs, thereby visualizing the fluorescence changes. In the absence of target cells (ECs), the fluorescence of the LV-EcpG biosensor was so weak that it was almost undetectable, indicating that the sensor was in an inactive “off” state. This state helped to reduce background noise. However, once a target cell (ECs) was introduced into the system, the situation changed dramatically; the addition of ECs triggered the binding of E12P to ECs, which led to an energetic change that was sufficient to activate the chromophore of the fluorescent protein, allowing it to emit a stronger fluorescence. This change in fluorescence intensity was a clear sign that the biosensor had been successfully activated, indicating that the sensor had shifted from an “off” state to an “on” state (Figure 1c). This study demonstrates an innovative and refined strategy aimed at developing a real-time detection tool against ECs with a genetically encoded FP combined with specific affinity peptides.

### 3.2. Characterization of LV-EcpG Biosensor in Cultured Cells

**Figure 2:**
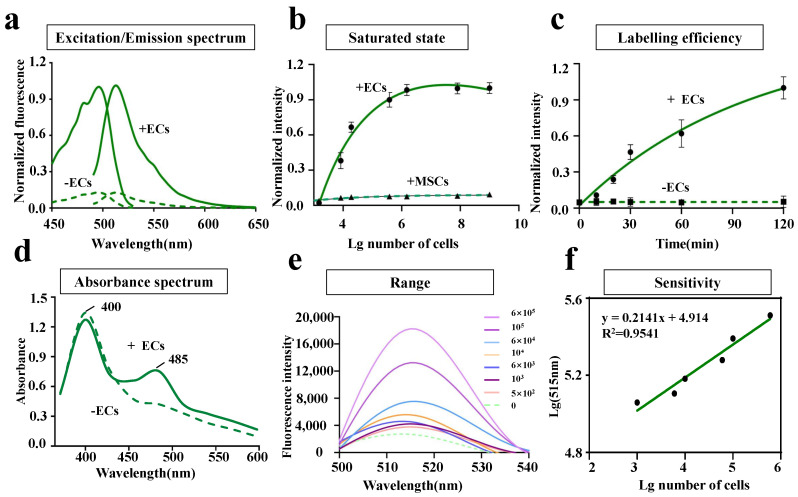
Characterization of LV-EcpG biosensor performance. (**a**) The excitation and emission spectra of 5μM LV-EcpG biosensor in the presence (solid line) and absence (dashed line) of $10^{6}$ ECs. The FI was normalized to the maximum without target cells. (**b**) Dose-response curve of LV-EcpG biosensor combined with different numbers of ECs and MSCs. Lines were best fits of the data to the one-site binding equation, and all data are normalized to the initial value. ECs quantity/100 μL. (**c**) Fluorescent labeling efficiency of LV-EcpG biosensor. Fluorescence was activated in EC manner with half-times 0.36 h. Lines were best fits of the data to a single exponential function, and all data are normalized to the maximum FI in the absence of ECs. (**d**) Absorbance spectra of 5μM LV-EcpG in the presence (solid line) or absence (dashed line) of $10^{6}$ ECs. (**e**) Fluorescence spectra of LV-EcpG in different numbers of ECs. EC quantity/100 μL. (**f**) Linear relationship between lg 515 nm and lg numbers of ECs. Data are plotted as mean ± s.e.m and, in some cases, are smaller than the symbols used for the mean. (*n* = 3 technical repeats, defined as data obtained from the same stock of purified protein).

To investigate the properties of LV-EcpG in vitro, the protein of LV-EcpG was expressed and purified using *Escherichia coli* (*E. coli*) at 37 °C. As anticipated, LV-EcpG predominantly exhibited low or no fluorescence in the absence of ECs binding. This phenomenon was primarily attributed to the immature (but protonated) state of the FP chromophores [22]. After introducing the LV-EcpG biosensor into the EC culture, its excitation and emission peaks were detected to be 495 nm and 515 nm, respectively, similar to the spectral distribution of GFP. Its excitation peak was red-shifted by 3 nm, and the presence of ECs was 800% higher than the value of the absence of ECs (fluorescence intensity) (Figure 2a). Moreover, an absorption spectra of 5μM LV-EcpG in the presence and absence of $10^{6}$ ECs revealed a decrease in the absorption peak at 400 nm and an increase in the peak at 485 nm (Figure 2d). These data suggested that the LV-EcpG biosensor altered the protonation/deprotonation ratio of its chromophore when bound to ECs, resulting in changes in fluorescence intensity.

In a comprehensive investigation of the efficacy of LV-EcpG on ECs, the emission fluorescence spectra of 5μM LV-EcpG in varying numbers of ECs were measured (Figure 2e). It was observed that the fluorescence intensity at 515 nm gradually increased with the number of cells. Moreover, an excellent linear relationship was found between the number of cells and the fluorescence intensity at 515 nm, with a linear coefficient of 0.9541, indicating a high sensitivity of LV-EcpG to ECs (Figure 2f). The dose-response curves of LV-EcpG for ECs and MSCs (Figure 2b) revealed significant results regarding the affinity and potency of LV-EcpG for ECs. This analysis showed that the limit of detection (LOD) was approximately 0.3 × $10^{4}$ cells per 100 μL of liquid, and when the cell count reached 6 × $10^{5}$ cells, it was considered the highest detectable amount of LV-EcpG. This indicated that LV-EcpG was effective even at low cell counts. Our innovative EC detection method made significant progress in methodology compared to the existing methods. There is currently no specific report on the LOD value of cell marker antibodies. Due to differences in detection methods, experimental conditions, instrument sensitivity, and sample processing, it is difficult to provide a unified and specific value. By fitting the data to Hill’s equation, a dissociation constant $K_{d}$ value of 0.216 ± 0.089 and a Hill coefficient of 6.288±0.54 for ECs was obtained at 37 °C. Meanwhile, MSCs values cannot fit the curve. This indicated a high affinity between LV-EcpG and ECs, and the effective interaction between LV-EcpG and ECs was crucial for their biological activity. Additionally, the instantaneous increase in fluorescence intensity at different time points was fitted to a single exponential function (Figure 2c), revealing an activation rate $k_{on}$ of $1.6\pm 0.46\;h^{-1}$ at 37 °C. This value was lower than the previously reported maturation rate of GFP $\left(0.8\;h^{-1}\right)$ [23]. Previously, there was no definitive determination of the maturation rate for this class of truncated fluorescent proteins, but the available data suggested that $k_{on}$ (association rate constant) may have been partially limited by the conformational change step triggered by binding to the target via the input structural domain prior to the maturation reaction of the chromophore. Overall, these results indicated that fluorescence activation of LV-EcpG was highly dependent on its high specificity in targeting ECs.

**Figure 3:**
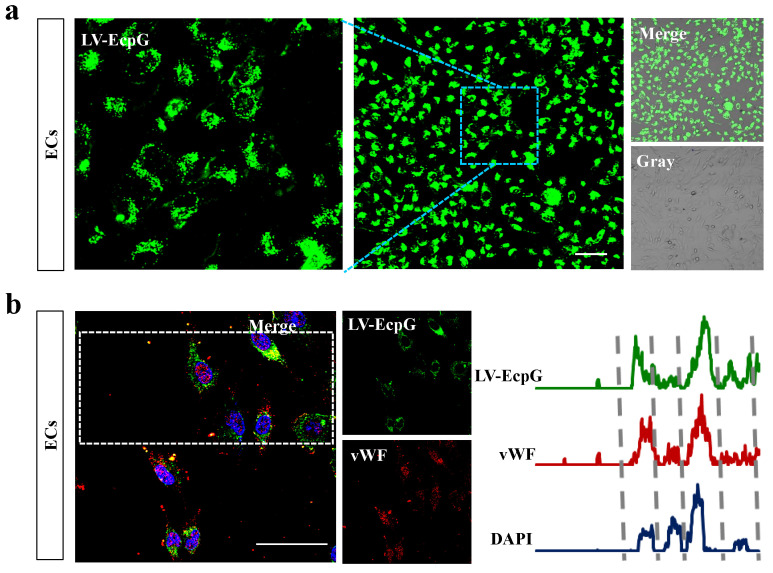
Confocal image of LV-EcpG biosensor. (**a**) Representative fluorescence image following the incubation ECs with LV-EcpG. (**b**) Co-localization of LV-EcpG and vWF in ECs were depicted through representative fluorescence images (left), with statistical analysis of fluorescent co-localization traces (right), delineated by dashed lines for each cell. *n* = 3 technical repeats, Scale bars, 40μm.

**Figure 4:**
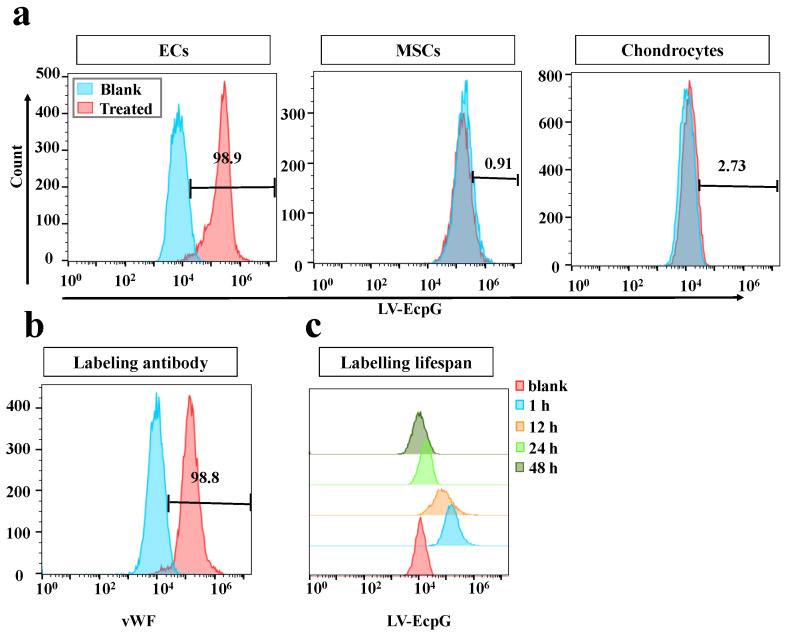
Flow cytometry detection of LV-EcpG biosensor performance. (**a**) Histogram of flow cytometry fluorescence distribution of LV-EcpG with ECs (left), MSCs (middle), and chondrocytes (right). (**b**) Flow cytometry histogram of vWF expression in ECs. (**c**) LV-EcpG biosensor fluorescence lifetime histogram. Histograms of fluorescence intensity distribution for flow cytometric analysis of LV-EcpG labeled ECs at different culture times. Data are plotted as mean ± s.e.m and, in some cases, are smaller than the symbols used for the mean. (*n* = 3 technical repeats, defined as data obtained from the same stock of purified protein).

We used the strategy of directly introducing the LV-EcpG biosensor (50μM) into the EC culture system and performing a 1-hour incubation. Afterwards, confocal microscopy fluorescence images showed that the LV-EcpG labeled the target cells with an efficiency of 97% (Figure 3a), demonstrating its excellent labeling ability. This showed that LV-EcpG predominantly interacted with the surface of the ECs, exhibiting minimal intracellular localization, a mechanism reminiscent of phage–cell interactions [24]. This remarkable binding specificity was attributed to the high-affinity peptide E12P, which was identified through phage display technology, enabling targeted recognition of EC surface markers. Parallel to this, a flow cytometry assay further confirmed this high efficiency, showing that the labeling efficiency of LV-EcpG was 98.9% (Figure 4a), and verifying its broad applicability in cell populations. In addition, we found that LV-EcpG could also label fixed cells, allowing for subsequent co-labeling with antibodies targeting specific EC biomarkers. This provided the possibility of multidimensional analysis. Specifically, single-cell tracking demonstrated that LV-EcpG co-localized with von Willebrand factor (vWF) antibodies in the same cells with a co-labeling efficiency of 99% (Figure 3b). This indicated that LV-EcpG not only matched the accuracy of antibody detection but also simplified the detection process. Meanwhile, flow cytometry detection of vWF antibody-labeled ECs exhibited an efficiency of 98.8% (Figure 4b), further reinforcing its reliability as a detection tool. To ensure the consistency and accuracy of the experimental results, all the cells used for testing were cultured under the same conditions and with the same passages. These findings indicated that the performance of the LV-EcpG biosensor in ECs binding not only achieved a precision comparable to that of traditional biomarker antibodies but also demonstrated an efficient detection capability, while simplifying the operation process and enhancing detection efficiency.

To further validate the specificity of the LV-EcpG biosensor, we introduced MSCs and chondrocytes as controls. The preliminary evaluation using a fluorescence photometer showed that the fluorescence intensity of LV-EcpG was approximately 4.5-fold higher in the presence of ECs than in the presence of MSCs or chondrocytes, and the latter two were similar to the culture medium control (Figure A1d). Subsequently, flow cytometry analysis further confirmed that LV-EcpG was negative for labeling of both MSCs and chondrocytes (Figure 4a), solidifying its position as a specific EC marker.

Given that pH was one of the key factors affecting the performance of the biosensor [25], we examined the pH response characteristics of LV-EcpG. By plotting the pH titration curve, the fluorescence intensity of LV-EcpG was observed exhibiting an increasing trend as the pH increased from 6.0 to 8.0 (Figure A1c), a finding that was crucial for optimizing the conditions of the biosensor in practical applications. Notably, the activity changes in LV-EcpG in practical applications were associated with its own fluorescence quenching but have no significant effect on cell growth and proliferation (Figure A1b). Furthermore, flow cytometry analysis revealed that the labeling rate of LV-EcpG decreased significantly with time, particularly after 48 h of sample treatment, when a negative fluorescence signal was detected (Figure 4c). This discovery suggests that LV-EcpG may possess the ability to self-degrade or be taken up by cells, which implies that it would not interfere with normal cellular functions or subsequent experiments. The intrinsic fluorescence quenching capability of the LV-EcpG biosensor offered novel opportunities for continuous multidimensional fluorescence detection, which was unachievable with traditional immunoassay methods. However, due to the disappearance of fluorescence after 48 h, if it was applied to long-term monitoring after, further exploration could have been conducted on the timing of multiple supplementary additions. Consequently, LV-EcpG could serve as a convenient and interference-free tool for multidimensional cell detection.

### 3.3. Visualization of the Directed MSC Differentiation Process into ECs

**Figure 5:**
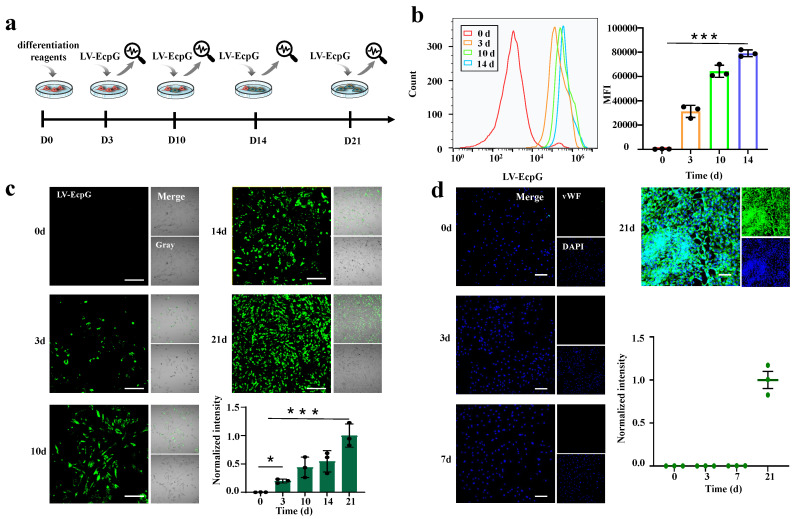
Visualization of the directed differentiation of MSCs into ECs. (**a**) Schematic of biosensor usage. (**b**) Flow cytograms (left) and the MFI histogram (right) of MSCs differentiated into ECs using LV-EcpG real-time detection. (**c**) Fluorescent representative images of MSCs differentiated into ECs using LV-EcpG real-time detection and histograms of the proportion of LV-EcpG. (**d**) vWF immunofluorescence images of MSC–EC differentiation at different time points and its statistical graph. Scale bars, 40μm. Each data point represents a visual field, * *p* < 0.05, *** *p* < 0.001, *n* = 3 technical repeats. Data were plotted as mean ± s.e.m.

MSCs could be induced to differentiate into endothelial-like cells under certain conditions, promoting angiogenesis in ischemic regions. Given this characteristic, MSCs have shown immense potential in tissue-engineering blood vessel construction, paving the way for new therapeutic approaches in ischemic heart disease. Therefore, it is particularly important to develop a tool that can monitor the real-time directional differentiation process of MSCs into ECs. To achieve this goal, this study attempts to use an LV-EcpG biosensor for the visual detection of ECs. EC culture medium (EBM-2) with 10 mg/L vascular endothelial growth factor (VEGF) and 5 mg/L basic fibroblast growth factor (bFGF) was used as the induction medium for MSCs to differentiate into ECs. At different differentiation time points, the purified protein of LV-EcpG 50μM was introduced into the cell culture medium during the differentiation process, and the change in fluorescence intensity was analyzed by the device, which allowed for the real-time visualization and tracking of the differentiation process of MSCs into ECs (Figure 5a). Through fluorescence imaging, we were able to clearly observe the dynamic changes in fluorescence distribution over time, providing a more accurate understanding of the cell differentiation process. On day 0 of differentiation, no fluorescence was observed. From day 3 to day 21, the green fluorescence intensity and distribution rate of LV-EcpG gradually increased over time (Figure 5c). Notably, during differentiation, we could observe that the distribution of fluorescence within individual cells progressively increased with time. This phenomenon highlights the gradual and subtle nature of the differentiation process. Meanwhile, for comparison, we detected the expression of the endothelial cell marker vWF during the differentiation of MSCs to ECs using the conventional immunofluorescence technique (Figure 5d). LV-EcpG marker detection exhibited significant fluorescence on the 3rd day of differentiation, while antibody vWF was only detectable on the 21st day. The observation results indicated that the LV-EcpG detection mechanism was more consistent with the complex and delicate slow differentiation phenomenon of stem cells [26,27]. These findings suggest, in particular, that our LV-EcpG biosensor has a higher efficiency than conventional methods because it can detect differentiation at an early stage.

Additionally, we employed flow cytometry to examine the differentiation of MSCs into ECs at 0, 3, 10, and 14 days, as labeled by LV-EcpG (Figure 5b). Over time, the fluorescence-labeled histogram shows that the population of fluorescence-labeled cells gradually increases. Quantitative analysis of the median fluorescence intensity (MFI) also corroborated this time-dependent shift, with the LV-EcpG increasing from 143 to 78,682. These findings are consistent with the fluorescence imaging results in Figure 5c. In summary, our experiments indicate that LV-EcpG enabled the real-time visualization and monitoring of MSCs differentiating into ECs at the level of living cells.

## 4. Conclusions

LV-EcpG combines the fluorescent reporter protein EGFP with the highly specific peptide E12P, which represents an innovative tool for the highly sensitive and specific detection of target ligands, particularly ECs. Some studies have reported screening affinity peptides through phage display technology, then binding them with FP (binding domains fused with FP [28]) or fluorescein to label the target [29,30]. Compared to them, the LV-EcpG biosensor boasts a unique “on” and “off” switching mechanism that enhances the detection precision by ensuring that the fluorescence signal only exists when ECs specifically bind. In addition, fluorescein may interfere with the normal physiological functions of cells and even lead to cell death [7,31,32]. Therefore, multiple toxicity tests and the optimization of experimental conditions are required. Notably, the stability and efficiency of LV-EcpG labeling must be optimized for long-term use. The future research should address these aspects to ensure the reliability and effectiveness of biosensors when applied in clinical settings.

The single FP characteristic of LV-EcpG offers flexibility in multicolor combined detection, allowing for the simultaneous monitoring of multiple biological events within cells when paired with other fluorescent tags. This feature is instrumental in unraveling intricate intracellular signaling networks and biological processes. The development of the LV-EcpG not only introduces an innovative tool for ECs detection but also presents a novel strategy for constructing fluorescent biosensors targeting complex targets, including cells and macromolecules. The novel strategy is pioneering and multi variable, and researchers can achieve their goals with minimal improvements based on their needs, such as replacing the tag protein EGFP with near infrared fluorescence or replacing the target binding domain (E12P). In summary, the LV-EcpG biosensor represents a significant advancement in the field of cell detection; is suitable for various devices such as confocal microscopy, flow cytometry, and fluorescence enzyme-linked immunosorbent assay; and has the potential to become a new alternative to antibody-based EC detection. It has promising applications in the commercial cell detection of endothelial cells, in vivo tracking of endothelial cells, and cardiovascular tissue engineering in the future.

## Data Availability

Statistical analyses (excluding sequencing data) utilized GraphPad Prism software (version 8.0). Gaussian-distributed data underwent either a two-tailed unpaired Student’s *t*-test (for two groups) or a one-way analysis of variance (ANOVA) with Tukey’s multiple comparison test (for multiple groups). Each quantification relied on a minimum of three independent experiments. Data collection and analysis were not blinded to experimental conditions, and no statistical methods were employed to predetermine sample size, which was based on prior experimental knowledge. Sample sizes used for statistics are indicated in Figure titles, with no exclusions from analysis. Sequencing data underwent standard procedures and software analysis, with significance levels displayed in each graph.
